# Teaching Gerontology in Transcultural Academics: A Phenomenographic Study of Thai and Swedish Nurse Educators’ Conceptions of Gerontological Nursing

**DOI:** 10.1177/10436596211068432

**Published:** 2022-01-08

**Authors:** Karin Mattsson, Sirpa Rosendahl

**Affiliations:** 1 Karolinska Institutet, Stockholm, Sweden; 2 University of Skövde, Skövde, Sweden

**Keywords:** aging, cultural competence, gerontological nursing, long-term care, nurse educators, qualitative, transcultural

## Abstract

**Introduction:**

There is an urgent need for registered nurses with gerontological competence within long-term care (LTC) of older adults. Despite increases of life expectancy, LTC for older adults is not emphasized in nursing curricula in neither Sweden nor Thailand. Thus, the aim was to explore conceptions on gerontological nursing (GN) among Swedish and Thai nurse educators.

**Method:**

A qualitative phenomenographic method, based on open-ended interviews with five Thai and nine Swedish nurse educators was conducted.

**Results:**

The results indicate a paradox between the educators’ knowledge about the implications of global aging, their hope of own aging, and LTC. The ethical responsibility of being credible and a source of inspiration in teaching about aging are focused, while GN seem to be less important.

**Discussion:**

To increase students’ interest in GN, measures need to be taken within the educational arenas, where the educators’ own conceptions toward GN, cultural aspects of aging, and LTC are discussed.

## Introduction

For a long time, there has been an urgent and growing need for registered nurses (RNs) with competence in gerontology. This has been thrown into sharp relief by the COVID-19 crisis of 2020 (Organization for Economic Co-operation and Development [OECD], 2020a). Gerontological nurses work in collaboration with older adults, their families, and communities to support healthy aging, maximum functioning, and quality of life, as well as recognizing and addressing illnesses and conditions that often occur in aging (Touhy & Jett, 2014). Gerontological nursing (GN) thus includes competence in geriatric, preventive, palliative, and culturally sensitive person-centered care (Rosendahl et al., 2019; Tassone Kovner et al., 2002). The concept of person-centered care, based on the individual person’s values, preferences, and symptoms, has been adopted by policymakers to empower patients and improve the quality of care (American Geriatrics Society [AGS] Expert Panel on Person-Centered Care, 2016; Jones, 2011; Moore et al., 2017). In the international arena, person-centered care is included in nursing students’ curricula, but nursing education programs are not “gerontologized” enough to ensure graduates have the competences to meet the needs of an aging population (Touhy & Jett, 2014). To further the development of person-centered care for older adults, as well as to recognize and address illnesses, gerontological nurses are best placed to promote education and training of other staff, such as nursing assistants or family members caring for an older person at home. The care provided by nursing assistants focuses on giving the older persons extensive support in daily life, such as helping them with dressing, eating, and personal hygiene (Holmberg et al., 2019).

Earlier studies have reported that nursing students have negative attitudes toward clinical placements and working within care of older adults as it is considered to offer limited learning opportunities (Abrahamsen, 2014; Øster et al., 2017; Lea et al., 2017). Various measures, such as public image campaigns and improving working conditions, have been taken to increase retention and students’ interest in long-term care (LTC) of older adults (OECD, 2020b). Studies have also shown that formal education in gerontology, combined with personal encounters with older people, has positive implications for how older adults are regarded and treated by younger generations and health care professionals (Lee et al., 2018; Mattos et al., 2015; Mattsson & Pietilä Rosendahl, 2017). Much of the focus has been on student and nurse perspectives, while this study explores nurse educators’ conceptions of GN in two different cultures, with similar challenges in increasing nursing students’ interest and competency in LTC of older adults with compromised health. Older adults refer to persons above the chronological age of 60 to 65 years with a functional, physical, and/or mental decline which imply the need for LTC and support from formal carers. This age span is equivalent to the retirement age in most nations and what the World Health Organization (WHO, 2018) refers to when describing global aging.

## Background

Driven by falling fertility rates and increases in life expectancy, the aging of the world’s population will continue and accelerate. This will have challenging economic and fiscal consequences despite a proposed higher Social Security retirement age (UN, 2019). Living longer may also increase the proportion of remaining life with compromised health, disability, and an increased need for LTC (Robine, 2021). In several of these countries with a rapidly aging population, such as Thailand and Sweden, migration has become a major component of population change. Between 2010 and 2020, Europe has been among the regions receiving the most migrants, whereas other regions such as East Asia and parts of Africa are net senders (United Nations [UN], 2019; Vollset et al., 2020). Migrant care workers have been described as the solution to the staffing crisis within LTC of older adults, but the challenges associated with multicultural care and workplaces are less discussed (Torres et al., 2014). Work associated with the care of older adults is undervalued, underpaid, and under-resourced globally (OECD, 2020b). In Swedish media, it is assumed that the kind of work the LTC-sector offers is not attractive and thus inconceivable for young, ambitious, ethnic Swedes (Szebehely et al., 2017; Torres & Lindblom, 2020).

In Sweden, where 20% of the population is 65 years and older, formal care and social support have been developed according to the Nordic welfare model (Swedish Institute [SI], 2021). However, during the last decades, Sweden has experienced a dip in its levels of tax-funded home care following restrictions at the municipal level (Centre for Policy on Ageing [CPA], 2014). Older adults with comprehensive and complex care needs now remain in their own homes, receiving care and support from family, home care assistants, and district nurses from primary health care centers or the municipality (Gransjön Craftman et al., 2018). The number of places in Swedish nursing homes has decreased in line with the national policy of supporting older persons to live at home for as long as possible, and for those who are eligible to move into a nursing home one third die within a year, indicating that they are in a palliative phase by the time they are admitted (Meinow et al., 2020; National Board of Health and Welfare [NBHW], 2019). Due to the shortage of RNs working within the care of older people, the work of nursing and home care assistants includes both social care and advanced health care tasks. For example, these assistants administer prescribed medication and manage clients’ palliative care even though they lack the professional support and basic training for those tasks (Holmberg et al., 2019). As the study by Gransjön Craftman et al. (2018) describes, nursing and home care assistants possess insufficient knowledge when changes in a patient’s condition occur, especially considering their key role in alerting health care professionals such as RNs or emergency medical staff.

The Asia Pacific region is aging more rapidly than many other parts of the world (Barber & Rosenberg, 2017), and Thailand is considered a “super aging” society, where the share of the population aged 60 years and above, 20% in 2021, is projected to reach 28% by 2031 (The Government Public Relations Department [GPRD], 2020). An aging population alongside socioeconomic developments, migration, and urbanization is eroding the traditional familial system of old age support (Knodel et al., 2013). Thus, in the National Plan for Older Persons, the government of Thailand has begun to include strategies for LTC provisions which cover activities promoting and supporting informal care within the family, providing health and social services in home/the community and institutional nursing homes (WHO, 2020). The universal coverage scheme in health care, implemented since 2002, is supposed to finance formal LTC for older adults in the future. Efforts are also being made to increase the number of health personnel with a specialization in gerontology and geriatrics. Because these disciplines are not popular, incentives in the form of scholarships and rewards have been suggested by the government (Knodel et al., 2015). RNs and advanced practice nurses (APNs) are best suited to give support to and educate formal and informal carers, as many older adults in Thailand live with chronic diseases and have limited access to prevention and basic control of health (Prajankett & Markaki, 2021).

Increasing the number of nursing graduates who are interested in caring for older adults is a challenge. Nurse educators play an important role in promoting students’ interest in GN but studies have shown there is insufficient gerontological and geriatric content in current nursing curricula, as in all health sciences educational curricula despite clear recognition of need (Bardach & Rowles, 2012). According to Garbrah et al. (2017), the lack of positive experiences with older people before and during their studies may prevent nursing students from choosing GN as a career option. Pedagogical strategies are crucial to improve education and nursing students’ communication skills with older adults.

Moreover, nursing faculty and clinical mentors/supervisors need to show interest, enthusiasm, and address issues from a transcultural perspective (Fagerberg & Gilje, 2007; Garbrah et al., 2017). This is important in fostering positive attitudes; the process of education can otherwise consolidate rather than dispel ageism (McLafferty, 2005). In their literature review, Millns Sizer et al. (2016) found that faculty can exert both an overt and covert influence on students’ attitudes toward caring for older people. If nursing students are given the impression that working with older people would be “wasting their education” or that caring for older people “is lacking in challenge” (Millns Sizer et al., 2016), working in LTC will not be their first choice of career.

According to Fagerberg and Gilje (2007), transcultural nursing research can add to the breadth and depth of curricular decisions and help address the cultural aspects of caring for older adults that need to be reflected in nursing curricula. This would better prepare students and future RNs to meet the needs of an aging population. Aligning nurse education with the changing realities facing nursing practice, where intercultural teams are caring for culturally and linguistically diverse older adults and their families, is essential (Markey et al., 2021).

As faculty have an influential position in regard to students and curricula, it is important to study the educators’ attitudes toward the field of GN. The aim was thus to explore conceptions on GN among nurse educators, who in this study came from Thailand and Sweden. As described above, Sweden and Thailand are representative of the similar challenges facing these regions both demographically and in nursing education.

## Method and Materials

This is a sub-study in a larger research project, “Teaching gerontology from a Global Perspective.” For this study, a qualitative method with a phenomenographic approach was used. Phenomenography investigates the qualitatively different ways in which people conceive various phenomena and is also useful when it comes to exploring approaches to teaching, and outcomes of learning in higher education (Marton, 1988; Sjöström & Dahlgren, 2002). According to Stenfors-Hayes et al. (2013), phenomenography is not only intended to explore how a phenomenon is conceived; it is also a method for examining how the ways of understanding are related to one another. Marton (1981) suggests that within phenomenography, there is a distinction between “what something is” and “how it is perceived.” “What something is” is referred to as the first order of perspective where the focus is directed toward the phenomenon itself, that is, the facts about what is observed. The second order of perspective, “how it is perceived,” focuses on how participants perceive a phenomenon in their own individual reality.

### Participants

Interviews were conducted with teachers and program directors in Swedish and Thai Baccalaureate nursing programs. Participants were chosen strategically according to teaching position, age, and gender to capture a variety of nurse educators’ conceptions on GN to explore the role that the teaching of GN has in curriculums. All educators who were approached, nine Swedish and five Thai, agreed to be interviewed. Of the 14 participants, 11 were employed as a nurse educator/senior lecturer; three of those were also directors of their department and one held a position as a career consultant in nursing. Two were program directors of nursing study programs, one at a Swedish and the other at a Thai university. Four of the Thai nurse educators, who came from four different universities, were doing part of their doctoral studies in Sweden at the time of the interviews. One program director was interviewed at a university in Thailand by a Thai colleague. The Thai nurse educators were all, except for one department director, doing clinical bed-side teaching and supervising as well as theoretical teaching in Thailand during their doctoral education. The role of an educator at a Swedish university involves only theoretical teaching and a joint assessment of students’ achievements during clinical placements together with the student’s clinical supervisor. One Swedish nurse educator was combining academic work with work as an RN in municipal LTC of older adults. The other nurse educators’ gerontological knowledge was based on earlier working experiences, personal relationships with older relatives and friends, and on reading literature out of self-interest. The age range of the participants was 35 to 64 years. There were 12 females and two males (one Swedish and one Thai). None of the participants had undergone specialist education in gerontology or GN.

### Study Context and Procedure

The study was held at a Swedish university with intense international educational and research collaboration with Thai universities within the nursing discipline. Individual interviews were conducted and audio recorded at the educators’ workplace. Open-ended questions—were asked (see Appendix). The nine Swedish participants were interviewed in Swedish and four Thai participants in English by authors K. M. and S. R. A fifth Thai participant was interviewed in Thai by a Thai colleague who is fluent in both Thai and English. All interviews, which were between 30 and 45 min long, were transcribed *in extenso* to constitute the final material for the analysis.

### Data Analysis

The interviews were analyzed using phenomenography in seven steps as described by Stenfors-Hayes et al. (2011, 2013). First, the transcribed interviews were reviewed independently by both authors and read through several times for inter-rater reliability. The second step involved searching for distinctive characteristics regarding the respondents’ views on GN. The third step included condensation to identify significant examples of the respondents’ conceptions. Fourth, the findings were compared and grouped into categories according to similarities and differences. In the fifth and sixth steps, the categories were articulated and labeled to highlight the most significant content. The content in the labeled categories was discussed by the authors and rechecked to ensure that each category was organized around a unique core. The seventh step involved contrasting, that is, a comparison of the categories and an additional check regarding the aim of the study, namely, to explore conceptions of GN among nurse educators. In the beginning, all transcripts were treated as a whole, rather than 14 different sets of data. After the main analysis, the responses were analyzed also on an individual level. The above steps were repeated several times.

### Ethical Considerations

The study was approved by the regional ethical review board at Uppsala University, Uppsala, Sweden (Dnr. 2015/322) and School of Nursing Science, Rangsit University, Thailand (ID 59 361-001). Participants were ensured confidentiality and were given written and oral information about the purpose of the study and their right to withdraw without explanation. Participants gave their oral and written consent to participate in the study. Transcripts are stored in the authors’ password protected computers; no unauthorized persons have access to the data.

## Results

In this section, the three categories of Swedish educators’ (SE) and Thai (TE) nurse educators’ conceptions on GN are presented. The categories are related, but regardless of cultural origin there is a clear paradox between the nurse educators’ personal and professional views on aging and LTC of older adults and on including more content on aging in curricula. In the first category, *A Paradox Between Hope and Knowledge—*“*I’m Going to be the Lucky one*,” the educators referred to their personal relationships with older family members and discussed what might be expecting themselves as older adults and own frights and hopes regarding aging. In the second category, *Long-Term Care of Older Adults—a Paradox Between Knowledge of Morbidity and the Conception of the RN’s Involvement*, the educators focused on cultural aspects of person-centered care and the competence of staff in LTC of older adults, rather than on the RNs function as a safeguard of monitoring the older adults’ health and illnesses. In the third category, *Teaching Nursing of Older Adults from Educators’ Pedagogical Perspective*, the educators focused on the ethical responsibility of teachers to inspire students and stimulate interest in GN.

### A Paradox Between Hope and Knowledge—“I’m Going to Be the Lucky One”

The core of this category is that older family members, especially grandparents, to a high degree shape the Swedish and Thai nurse educators’ personal conceptions on the need of nursing care in aging. The nurse educators talked positively about their past relationships with grandparents, whom they did not perceive as being old, though some of the grandparents were centenarians. They did not reflect on the grandparents’ aging until they needed assistance because of illness and functional decline. The thought of the educators’ own aging was daunting when illnesses were frequent in the family history. “Mom died in ALS [a progressive nervous system disease]; dad is 81 and multi-sick. He says he is old . . . it is sad to grow old and not be able to move and . . . alone . . .” (SE1). As young, the educators either relished being close to old family members or found older adults repulsive and aging intimidating:

*I feel warm when I am close to old people. It is like we are . . . mm . . . dependent and independent at the same time. Yes, the older persons that I live with, they take care of me . . . and at the same time, I take care of them too.* (TE7)

One’s own aging, later life, and becoming in need of care as old was something the educators normally did not want to think about. “I have this picture in my mind that it’s other people who are old, not me . . . That’s some old ugly guy who walks with a stick and sits on a couch drinking coffee” (SE4). Some educators with multimorbidity in the family history tackled their own aging by trying to live a healthy life. “My mother got type 2 diabetes, I don’t intend to get it, so I move more and eat no sugar. My husband gets very tired of me, I am constantly planning our aging” (SE2).

Aging is perceived as a process which has become apparent in the educators’ own existence from bodily sensations, lack of strength, mobility, and pain. Now middle-aged, the educators also described an awareness of psychological aging which gives new perspectives on life, “I can feel a little joy in getting older and feel a little more secure as well” (SE7). Aging is a transition into the unknown, “the more physical limitation I have, my interest in spirituality seem to grow” (TE13). But being dependent on care and assistance was not included in the educators’ own picture of the future. The hope was instead on belonging to those who enjoy life until the end, “I hope I can live until I die” (SE2). While Swedish educators envisioned their old age as healthy retirees, Thai educators’ vision of their own aging included continuing doing academic work and teaching. Taking care of their physical health in aging was equally important for Thai and Swedish educators. Thai educators also emphasized other aspects, like moral integrity and the importance of being revered, “for me, social participation is important, as an old man you cannot live alone, we must be respected by others and live morally” (TE9). As Thai academics, they can adopt a lifestyle which implies independency. For the vast majority, life as an older adult in Thailand is far more restricted because they either must assist or rely on assistance from family. Traditionally, and according to Buddhist principles, older people are highly respected but because of socioeconomic changes, this is eroding.

### LTC of Older Adults—A Paradox Between Knowledge of Morbidity and the Conception of the RN’s Involvement

This category includes educators’ knowledge of media reports about the challenges of an aging society, and their own experiences from being a part of a family member’s trajectory into dependency. Some of the educators had experiences of family caregiving for an older adult in LTC and next of kin of the older adult moving into nursing home. Based on their own experience or assumptions, most Swedish educators claimed that the LTC of older adults is neglected, has insufficient resources, and staff with inadequate knowledge. The view was that there is not enough focus on individual preferences or person-centered care, “when moving to a nursing home there is no saying of your own” (SE5). A comparison was made with how older people are regarded and cared for in other cultures, where the young are thought to care about and feel gratitude toward older family members in a higher degree than in Western societies. “In the future, I hope that LTC will improve . . . with more resources and that something will be done about the loneliness among older people” (SE12).

New technologies and changing ways of life are perceived as factors making older people in Thailand vulnerable and lonely. They take care of grandchildren and sometimes support adult children for as long as they can, but the younger generation do not care in the same way about the older people. “The gap between generations . . . sometimes the older people don’t understand the younger ones, and the younger ones don’t understand older people. The problems will increase I think” (TE9). In Thailand, socioeconomic growth, rapidly increasing life expectancy, and low birth rates imply a need for LTC plans for older people and age-friendly housing:

*In general, in Thailand—there is only one child per woman, which is a problem in the future—the family won’t have time to take care of old relatives. With not enough nursing homes . . . families have no choice. It’s a big problem.* (TE9)

All the educators were aware of the ongoing demographic changes in society. They all reflected on the problems associated with managing an aging population in the future. Despite this, none of the educators stress the RNs role in monitoring the older persons’ health within LTC, where the vast majority of older adults will live with comorbidity and advanced health conditions for several years until death. “They [older adults] are the ones with medical problems. It is other staff that take care of those who need support and service . . . It is not the RN” (SE7). Only the Swedish educators reflected on the need for nurses in LTC to possess cultural awareness and cultural sensitivity. They also talked about immigrant health care workers having an inherent caring attitude toward older adults. “They were absolutely fantastic in their care and treatment of the elderly—they show more respect” (SE1). An RN in municipal care is responsible for supervising the nursing care provided by a multicultural team of nursing and care assistants, whereas the majority of the older population in Sweden is still predominantly people born in Sweden:

*We are lucky to have many new Swedes coming from other parts of the world who can help us deal with the situation of a growing number of older people—But [working with older adults] requires a knowledge of the context you are in and the country's history. It is not enough simply to know how to give nursing care.* (SE5)

Not only does the RN in LTC require skills in leadership, personal characteristics such as problem-solving ability, courage, and high ethical standards are also essential by some educators. The RN working in LTC of older adults must be firmly anchored in evidenced-based nursing and in line with legislation to secure patient participation and safety. Some educators reflected on the advocacy of nurses, their duty to react and defend the older person’s integrity and dignity if threatened or violated by members of staff or by family members:

*The personal responsibility of the nurse must be discussed. I mean when you see maltreatment of the older adult . . . What responsibility do I have if I notice it? Can I just say, no . . . someone else can take care of it? No, it’s not about anyone else, YOU must take responsibility for this.* (SE3)

The Thai educators reflected on the older population needing more preventive measures, health care, and LTC from professionals when staying at home. “In reality . . . we meet older adults in every area where we provide nursing practice” (TE13). With no former experience in caring for older adults, the professional start as a newly graduated nurse can be challenging. “I had no experience from either illness, disease, or older persons. It was a tough road to walk” (SE8).

### Teaching of Older Adults Does Not Include GN

The core of this category is that all educators were aware of the need of strengthening the education and content on care of older adults both in curricula and practical teaching, but none were mentioning the concept GN. Baccalaureate nursing programs are designed to give a generalist degree, but the educators reflected on students meeting older people in most health care facilities. The topics of aging and gerontology should be prioritized more, and not only in nursing education: “Architects and engineers also need knowledge about aging, gerontology” (SE4). Interdisciplinary courses were recommended by some of the educators, the higher education sector must adapt to the requirements of a changing and aging society where generations do not have natural places of interacting:

*It is VERY important for us to show the young students how to take care of older people. They don’t have enough knowledge . . . more than 50% of our students come from divorced families and are living alone or on campus. The parents only pay or send them money.* (TE9)

Theoretical education is not enough. In Sweden, LTC of older adults is viewed as being demanding, both in terms of cost and resources. As municipal economic resources have decreased, so has the possibility of giving person-centered care. Some of the educators reflected on the ethical stress this causes nurses, and on how this has implications when students are undergoing clinical education where they might encounter negative attitudes that put the students off from working as an RN in LTC. The shortage of RNs working within LTC implies that students, during their clinical education, are guided and supervised in basic care of older adults by staff not qualified and often lacking elementary and essential education on the tasks:

*There is a difference between learning something at school and encountering it in real life . . . to meet a person's need when they are completely dependent. And coping with anger and frustration, they can be very angry sometimes . . . but [as a professional] you must cope with that.* (SE1)

The cultural diversity among students is greater than among educators. The Swedish educators reported the percentage of students not born in Sweden to at least one third. Being culturally sensitive is not only about acknowledging that people come from different parts of the world but also about being genuinely interested, listening to the experiences of different generations, and being curious and aware of one’s own prejudices. Here, one of the participants makes an observation: “Aging looks different now, older adults are active and attend rock concerts and gyms” (SE8).

The educators also reflected on being credible as teachers of nursing students. If you teach something, your knowledge must be based on proven expertise and experience, genuine interest in the topic, and on current research. “We [as teachers] also need knowledge about aging and everything that aging include, from health needs, illnesses etcetera, but also . . . how do we deal with these older people? How do we take care of them?” (SE3). An educator, also being a program director, mentioned the national regulations regarding programs in the university sector as hindering innovative teaching and learning activities in GN. Assignments where students have personal encounters with older adults and are personally affected by someone or something could change negative attitudes:

And that, I think, is a core issue in education . . . lessons or assignments that are giving an emotional engagement. You can be transformed by being in a situation with someone. It is a way to develop as a human being. (SE5)

## Discussion

The findings of this study, with the aim to explore conceptions of GN among nurse educators, showed a similarity between Thai and Swedish nurse educators’ conceptions in terms of how personal relations and experiences are affecting their views of aging. The conceptions of aging and older adults seem to be more positive if the older persons in the educators’ past relations were in good health, but turns into a more negative view, when observing the decline of health and an increasing comorbidity. This is consistent with Jung and Jopp (2019) who argue that the quality of the relationship with one’s parents has an important role in shaping adults’ conceptions on aging in general and on one’s own aging in particular. Thai educators talk about a mutual dependency between generations, which might be a result of them living together with older generations during their childhood. The Swedish educators focus on continuing living in the same way as before. Becoming old, losing intellectual capacity, being dependent, and in need of care is frightening. For both Thai and Swedish educators, there is a paradox between their own hopes for the future and their knowledge about the normal implications of longevity, which often imply living with comorbidity, dependency, and in a prolonged palliative phase (Meinow et al., 2020).

In Thailand, the need for LTC arrangements of older adults is new and the concepts of gerontology and GN still unfamiliar within nurse education. The Swedish educators are familiar with the arrangement of formal care and social support of older adults, but only one had recent professional experience of working as an RN in municipal LTC and nursing homes, where the students have their clinical placements. Some of the Swedish educators’ statements might thus be colored by their own experiences of being next of kin of an older adult in LTC and by medias’ reports, more than on actual knowledge and experience in GN.

In the findings of this study, Swedish educators portray immigrant care workers as having an inherent caring attitude, innate caregiving skills, and deep respect for older people. However, they were not reflecting on their own cultural competency or the lack of diversity within the faculty of the university. This is consistent with the portrayal of immigrant workers in media as described by Torres and Lindblom (2020), where migrant care workers in “their otherness” are depicted as assets to the sector. They are portrayed as not only needing but also wanting, to take on Swedes’ caring responsibilities for older adults. In Stockholm County, 55% of the staff were born abroad (NBHW, 2019). The stigma of care work is also a stigma attached to ageism, which is not only noticeable in society. The “otherness” of becoming old is sometimes expressed by the older adults themselves, by their family and by staff in LTC facilities or home care (Dobbs et al., 2008). The COVID-19 pandemic has made ageism even more evident (Banerjee, 2020; OECD, 2020a; Skoog, 2020).

When they themselves become old, both Thai and Swedish educators want to be treated and regarded as unique human beings who have a say in their own life and care, and person-centered care is desired. Person-centered care requires caregivers who are willing and competent in engaging with the older adult, “such as learning about the person through life histories, listening to their life experiences, wisdom, dreams and fears” (Haugan et al., 2020, p. 9). Thus, person-centered care requires caregivers not only to be able to understand the older person’s language but also an interest in their social context and culture. Culture per se is not an issue discussed by the Thai participants, but because Thailand’s younger population is rapidly declining (Vollset et al., 2020), the need for incoming migrant care workers or technical solutions and care robots may soon be as urgent in Thailand as in countries such as Japan (Wright, 2019). In this study, Thai educators expressed concern about the future because the tradition of filial piety is eroding, and more young students live alone and come from divorced families. As an increasing number of students have not experienced close relationships with members of the older generation, societal and teaching strategies are needed to prevent negative attitudes toward aging and older people. Experiencing close personal encounters with older people, combined with formal education in gerontology, during which students gain an awareness of both the universality and uniqueness of aging, can foster positive attitudes toward caring for older adults (Marmstål Hammar et al., 2017; Millns Sizer et al., 2016).

More RNs qualified in GN are needed to relieve the heavy physical and psychological workload for the few staffed RNs in place (Bratt & Gautun, 2018; Kox et al., 2020; Marmstål Hammar et al., 2020). This has in Sweden, the rest of Europe, and in the United States become even more evident during the COVID-19 pandemic, which has also revealed an urgent need to increase the competence level of nursing and care assistants (Harrington et al., 2020; Kabir et al., 2020; OECD, 2020a). In municipal care and LTC facilities, the patient–nurse ratio greatly exceeds the standards which safeguard patient safety and improved health outcomes (Saville et al., 2019). In Sweden, the number of RNs in LTC has gone down since 2015, which stands in contradiction to the increased physical and mental health care needs (NBHW, 2012, 2019). When nursing students do clinical studies at LTC facilities where working conditions have deteriorated, this may worsen negative attitudes toward aging and diminish students’ interest in this sector as a future career path (Bratt & Gautun, 2018).

In this study, none of the educators had prior education in gerontology, but lack of qualified faculty with gerontological expertise within nurse education is not uncommon (Wyman et al., 2019). Nurse educators have an important role in promoting interest in GN and to challenge negative attitudes, cultural constructs, and stereotypes regarding older people (Davis & Smith, 2013). For nurse educators, the stigma of growing old is no less than in the rest of society. Also, ageist views and cultural preconceptions among nurse educators at universities may not be different than in society at large. Many of our attitudes are unconsciously internalized and might remain so until we are prompted to engage in reflection. According to Trigwell and Prosser (2004), there are links between the way teachers approach their teaching and ways their students approach their learning. When teachers adopt student-focused approaches to teaching, for example, by reflecting together on ethical aspects of students’ encounters with older persons, students can adopt a deeper approach to learning.

Accordingly, there are implications for heads of departments and academic leaders to consider and reflect on the competence development and composition of teaching staff, but also to raise their voices and practice leadership in the educational arena (Sing & Haynes, 2020), which include the clinical settings for students’ practical education. The COVID-19 pandemic has forced the education sector to make a rapid shift to distance teaching which implies changes in didactics. New models are needed to improve student learning of person-centered gerontological care that also address transcultural issues. An example is presented by Phillips et al. (2015) who propose an Ethno-Cultural Gerontological Nursing Model. By developing core competencies for GN, educators can demonstrate the values established by their academic institution and function as agents of change in promoting leadership, students’ interest, and organizational development in the care of older people (Wyman et al., 2019).

### Limitations

A limitation of this study is the small number of participants, but the material represents a variety of conceptions as the phenomenographic method suggests. As the concept of GN is still not used within nurse educations in Sweden and Thailand, and not addressed by name by the educators in this study, the researchers have checked, rechecked, and discussed the labeling of the categories several times. Another limitation is that LTC as a concept is not implemented in nurse education and is still relatively unknown among Thai educators. However, it is not necessary to have experienced a phenomenon to have conceptions about it. Instead, phenomenography focuses on what people think about a phenomenon.

## Conclusion

As there is a global shortage of RNs in all health care sectors, the question is how to make ends meet. Including more gerontological content in bachelor and specialist nursing curricula requires syllabi to be revised and audits to be made of the national regulating system of higher education. Because aging is both a universal and unique phenomenon and older people constitute a growing proportion of the global population who require increasing resources in health care, competence in GN is essential. In GN, person-centered care is inherent because individual wishes, cultural values, and preferences must be recognized. To increase students’ interest in GN, measures must be taken within the educational arenas. Further studies are needed to explore how the attitudes and interest of faculty are linked to students’ interest of further education or work within GN and LTC of older adults. Studies also need to review if the new methods of teaching imposed by the COVID-19 pandemic, using digital encounters with students can enable or complicate joint reflections on aspects of aging and patient-centered care where students and teachers also can be seen “Others.” Citing Torres et al. (2016), “can we really provide end-of-life care in a patient-centered and culture-competent way if we do not regard all patients and their families as potential ‘Others’ whose uniqueness we must make an effort to decipher?”

## Appendix

Interview guide with qualitative (open-ended) questions:
*What are your views on aging and older people?*

*As nurses, how can we give care and support to an older person?*

*What are your views regarding teaching GN within higher education from a national and international perspective?*
